# Intrusion detection system using Online Sequence Extreme Learning Machine (OS-ELM) in advanced metering infrastructure of smart grid

**DOI:** 10.1371/journal.pone.0192216

**Published:** 2018-02-27

**Authors:** Yuancheng Li, Rixuan Qiu, Sitong Jing

**Affiliations:** Department of Control and Computer Engineering, North China Electric Power University, Beijing, China; Beihang University, CHINA

## Abstract

Advanced Metering Infrastructure (AMI) realizes a two-way communication of electricity data through by interconnecting with a computer network as the core component of the smart grid. Meanwhile, it brings many new security threats and the traditional intrusion detection method can’t satisfy the security requirements of AMI. In this paper, an intrusion detection system based on Online Sequence Extreme Learning Machine (OS-ELM) is established, which is used to detecting the attack in AMI and carrying out the comparative analysis with other algorithms. Simulation results show that, compared with other intrusion detection methods, intrusion detection method based on OS-ELM is more superior in detection speed and accuracy.

## Introduction

To achieve dynamic charging capability, Advanced Metering Infrastructure uses Smart Meters(SM), two-way communication system, Home Area Network(HAN) and Metering Data Management System(MDMS) to establish communication links with users [1]. The communication process of AMI deploys a common communication protocol to meet the requirements of interconnection, which because the terminal devices of the client and part of the communication network are in an open form.

With the significant increase in the number of access points and detection paths, the probability of information security accidents will increase greatly due to the openness of information technology and the characteristics of users [2]. Computer malware represents a direct threat to the smart meter, through disconnecting the batch control switch, the blackout of numerous users will occur, and illegal users can steal information that records smart meter and electricity pricing information, even tamper the power data.

AMI computing, storage, and communication resources are limited, so it is not yet feasible that installing anti-virus software in the AMI and keep it updated [3]. Intrusion detection is based on certain rules or statistical analysis, which through collecting and analyzing audit records, security logs, user’s behavior, network packets, other information in the key points of the computer system and network to check the signs that are violated the security policy of the invasion or attacked in the network or system.

In order to ensure the security of AMI system, the academia, electric power operators and regulatory agencies have carried out information security research for the AMI system. NIST and Open Smart Grid put forward many research reports on information security [4]. Detection technology can monitor the running status of the system and discover various attack behaviors or attack results, which can effectively guarantee the confidentiality, integrity, and availability of system resources. In [5], a collaborative intrusion detection mechanism for AMI security is proposed and the constraint computing and storage resources of the smart meter are taking into account in the meantime. A new intrusion detection system framework based on data flow mining algorithm is proposed for the whole AMI system and it analyze the performance with an IDS dataset [6], which consists of three different levels of intrusion detection system. For the technology of intrusion detection, an intrusion detection model based on least squares support vector machine is proposed in [7], which uses commonly used information feature extraction algorithm to obtain linear and nonlinear dependent data features. In [8], a new hybrid intrusion detection learning model based on density, cluster center, and the nearest neighbor is proposed. In [9], a new detection method is proposed for network attack in the industrial control system. [10] divides the network traffic into multiple distributed intrusion detection systems to improve the detection rate of network attacks and balance the load of the intrusion detection system. A systematic approach is proposed to establish a hybrid intrusion detection system in [11]. The learning interval is based on the power system state specification, including normal control operation and cyberattacks, which employ a common-path mining technology from the synchronous measurement data and the system audit log mining related information, so establish the learning model accurately. Zhang et al. [12] uses the support vector machine (SVM) and artificial immune system (AIS) to detect and classify malicious data and possible cyberattacks. To deal with Distributed Denial of Service (DDoS) attack on the AMI network. [13] introduces honey into the AMI network as a decoy system to detect and gather attack information. Ntalampiras [14] proposes a novel methodology for automatic identification of integrity attacks and applies the approach to data coming from the IEEE-9 bus model, In addition, he proposed an anomaly-based methodology for reliable detection of integrity attacks in cyber-physical critical infrastructures in [15]. In [16–18], an overview of machine learning methods and the data mining algorithms in the intrusion detection system was provided.

The research of network intrusion detection technology is developing rapidly, existing work such as [6], [12], [13] is closely related to our work. However, Zhang et al. [12] focus on the intrusion detection in complete smart grid rather than aim at the security in AMI. Wang et al. [13] pay more attention to DDoS which is just one type of attack types, whereas we concentrate on performance and effectiveness with various of attack types in whole IDS. Zhang et al. did not give a specific comparison of used classifiers, which is outmoded. The algorithms used in our work such as ELM was proposed about a decade ago. The method which is proposed by [12] and [13] needs to set lots of training parameters artificially and leads to local optimum easily, in addition, [12] emphasized data-mining-based IDS using data stream mining in network layer in open systems interconnection (OSI) model and [8] specification-based IDS due to controlled network in AMI.

Which is characterized by the historical data in batches to train and support the number of samples can be changed, each round of training process training algorithm only enter the current batch of data and update the network weight, without duplication of historical data, generalization ability, taking into account the AMI system data will inevitably be data errors or missing cases, so the OS-ELM algorithm is more suitable for the needs of practical applications.

The main contributions of this paper are as follows: Firstly, sample and preprocess the data set, the method of gain rate evaluation is used to reduce the dimension of the data, and compared with other kinds of dimensionality reduction methods to verify its validity. Then, an intrusion detection system based on OS-ELM is proposed. A large number of experiments are carried out to validate the parameter selection using the current data set, and the parameters which are most suitable for the system model are determined. Finally, the OS-ELM detection algorithm is compared with other detection algorithms to verify the feasibility of the proposed method.

The remainder of the paper is organized as follows: The next section describes system model; the third section puts forward the OS-ELM based intrusion detection model; the fourth section analyzes and compares the experimental results; the final section contains a conclusion of the dissertation.

## The components and security analysis of AMI

### Components of AMI system

The structure of the smart grid includes four parts: Advanced Metering Infrastructure (AMI), Advanced Distribution Operation (ADO), Advanced Transmission Operation (ATO) and Advanced Asset Management (AAM) [19]. The AMI is a complete network processing system that includes measure, collect, store, analyze and information utilizes the user’s power consumption, which provides the communication and control functions for the smart grid [20]. In order to achieve fine-grained pricing mechanism, measure automation, demand response, promote quality management functions, promote two-way interaction between power system and users, and promote users’ rational use of electricity to provide technical basis.

**Fig 1:**
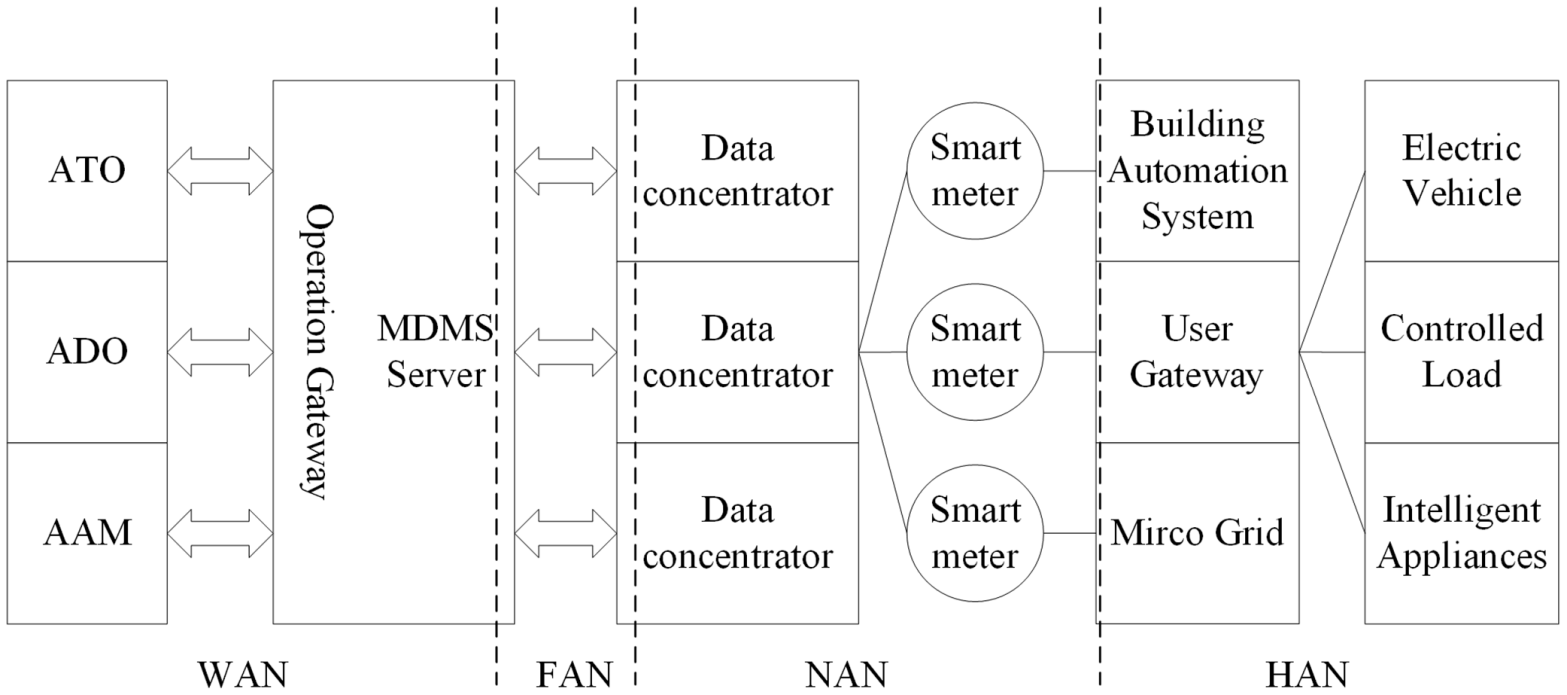
Structure logic diagram of AMI system.

AMI system consists of Smart Meter (SM), two-way communication system, Home Area Network (HAN), and Meter Data Management System (MDMS) [21]. The HAN enhances user’s experience of the electricity by adding the intelligent management unit. The metering data, which is collected by the smart meter, is sent to the MDMS via a bi-directional communication channel. The MDMS system is a database management system with an analysis tool that handles the measurement values store in the AMI database. Smart meters support instant reading and verify the user’s power consumption information, remote on and off, device interference and steal detection, time-of-use price, real-time price and other functions. The two-way communication network is AMI communications infrastructure that connects smart meters to MDMS. As shown in Fig 1:

### Security analysis of AMI system

AMI is mainly confronted with security issues, which are divided into two categories: the objective threat usually dues to the existence of communication systems or information system failures and the staff misuse; the subjective threat generally refers to a premeditated attack. There are various potential security problems on AMI as follows [22]:

Integrity security: data integrity is crucial in data delivery that between the sender and the receiver, since its violation not only can cause incorrect billing but also can launch malicious control commands towards AMI, which may result in a massive power outage.

Availability security: data availability threats will lead to buffer overflow, data loss on the collector side and cause a delay in data delivery, even data loss at the endpoints because of limited link bandwidth.

Common Network security: the types of threats include endpoint DoS, link flooding, wireless link jamming and so on.

Security issues for AMI in the smart grid have been widely studied. For example, many compromised collectors can launch DoS attack to headend [23]. Wireless communications in AMI are always threatened, so an AMI security framework based on information center network is proposed. The proposed framework can guarantee the stability and security of AMI system [24], The widespread deployment of AMI has had quite an opposite effect by fueling new ways to steal power and energy theft, which may cause enormous economic losses, [25,26] propose a detection mode which is mainly used to solve the power theft problem in AMI and the experimental shows that the model can detect various types of energy theft attempts accurately using individually inaccurate sensors. Under the background of continuously increasing traffic in AMI, finding a solution to meet the traffic requirements of AMI. [27–28] propose a public key cryptosystem security framework for AMI wireless network communication, which based on the creation of certificates and revocation of certificates to ensure system access security. [29] presents a layered specification-based IDS for HAN in AMI, paper defines specifications that extract from the IEEE standard as the normal behavior and the specifications deviations from the normal behavior can be malicious activities and we use the machine learning method to learn the characteristics of attack data. The intrusion detection problem can be as a two classification problem; we use of OS-ELM method for classification to achieve detection.

## Intrusion detection model based on OS-ELM

### The basic ideas and algorithm principles of ELM

Extreme Learning Machine (ELM) is a kind of generalized single hidden layer feedforward neural network (SLFNs). It uses the gradient-based learning algorithm to train the network, which is different from the traditional learning method and iteratively adjusting all parameters in the network. The traditional neural network needs to be set a large amount of training parameter when learning algorithm (back propagation algorithm) and it’s easy to produce the local optimal solution. For ELM, only the parameter of the optimal number of hidden units needs to be determined and assigns the input weights and hidden layer thresholds randomly. The output layer weights are calculated directly by the least squares method. The entire learning process does not require an iteration to complete, so it has been shown extremely fast with generalization performance better than the traditional learning algorithm. The ELM algorithm is described as follows:

Given*N* different sample {(*x*_*i*_, *t*_*i*_), *i* = 1, ⋯, *N*}, which *x*_*i*_ = (*x*_*i*1_, *x*_*i*2_, ⋯, *x*_*in*_)^*T*^ ∈ *R*^*n*^, *t*_*i*_ = (*t*_*i*1_, *t*_*i*2_, ⋯, *t*_*in*_)^*T*^ ∈ *R*^*m*^, the mathematical model of singe-hidden layer feedforward networks (SLFNS) with *L* implicit nodes is:

$$f_{L}(x_{j})=\sum_{i=1}^{L}\beta_{i}g_{i}(x_{j})=\sum_{i=1}^{L}\beta_{i}G_{i}(\alpha_{j},b_{i},x_{j}),j=1,2,\cdots ,N$$ (1)

Which *α*_*i*_ = (*α*_*i*1_, *α*_*i*2_, ⋯, *α*_*in*_)^*T*^ and b_*i*_ are the input weight and bias value of the ith implicit node respectively, *β*_*i*_ = (*β*_*i*1_, *β*_*i*2_, ⋯, *β*_*im*_) is the output weight of the connected with implicit node and output layer, *g*_*i*_(*x*_*j*_) = *G*(*α*_*i*_, *b*_*i*_, *x*_*j*_) represents the output *x*_*j*_ of the ith implicit node on the output.

If the actual output of the network is equal to the desired output, there is:

$$\sum_{i=1}^{L}\beta_{i}G_{i}(\alpha_{j},b_{i},x_{j})=t_{j},j=1,2,\cdots ,N$$ (2)

The above *N* equations can be written as matrices:

Hβ=T (3)

$$H=\left(\begin{array}{c} h(x_{1})\\ h(x_{2})\\ \begin{array}{c} \vdots \\ h(x_{N}) \end{array} \end{array}\right)={\left(\begin{array}{ccc} G(\alpha_{1},b_{1},x_{1}) & \cdots & G(\alpha_{L},b_{L},x_{1})\\ G(\alpha_{1},b_{1},x_{2}) & \cdots & G(\alpha_{L},b_{L},x_{2})\\ \begin{array}{c} \vdots \\ G(\alpha_{1},b_{1},x_{N}) \end{array} & \begin{array}{c} \;\\ \cdots \end{array} & \begin{array}{c} \vdots \\ G(\alpha_{L},b_{L},x_{N}) \end{array} \end{array}\right)}_{N\times L}$$ (4)

$$\beta ={\left(\begin{array}{c} \beta_{L}^{T}\\ \beta_{L}^{T}\\ \begin{array}{c} \vdots \\ \beta_{L}^{T} \end{array} \end{array}\right)}_{L\times m},T={\left(\begin{array}{c} t_{L}^{T}\\ t_{L}^{T}\\ \begin{array}{c} \vdots \\ t_{L}^{T} \end{array} \end{array}\right)}_{N\times m}$$ (5)

*H* is called the hidden layer output matrix, where the *i*th row represents the output of the *i*th input *x*_*j*_ with respect to the hidden layer, the *j*th column shows the output of all input *x*_1_, *x*_2_, ⋯, *x*_*N*_ with respect to the *j*th implicit node.

In the ELM algorithm, the input weight *α* and the bias *b* are randomly selected from a continuous probability distribution, so that the Eq (3) is a linear equation with variable *β*. Solve the linear system is equivalent to finding the minimum output weight $\hat{\beta }$ So that the error ‖*Hβ* − *T*‖ is minimized. Using the least squares method to calculate $\hat{\beta }$, the solution can be expressed as:

$$\hat{\beta }=H^{+}T$$ (6)

The steps of the ELM algorithm are as follows: Given a training data set Ω = {(*x*_*i*_, *t*_*i*_)|*x*_*i*_ ∈ *R*^*n*^, *t*_*i*_ ∈ *R*^*m*^, *i* = 1, 2, ⋯, *N*}, Activation function g: *R* → *R* and *L* implied points.

1. Randomly select the input weights *α*_*i*_ and the bias *b*_*i*_, *i* = 1, ⋯, *L*.
2. Calculate the hidden layer output matrix *H*
3. Calculate the output weights $\hat{\beta }=H^{+}T$, there *T* = (*t*_1_, *t*_2_, ⋯, *t*_*N*_)^*T*^.

### Principle and model of OS-ELM

Traditional ELM algorithm using batch learning model will begin a study after all data are transmitted to the system. However, it is practical that original data will arrive consecutively, and has no prior knowledge as to how many training observations will be presented. Therefore, taking into account the idea of online sequences and in [30] proposed online sequence limit learning machine (OS-ELM) to avoid the repetitive training through the method where only newly arrived data can be seen and the training observations are discard as soon as the learning procedure is completed. So OS-ELM is appropriate for intrusion detection in AMI.

Online sequence ELM algorithm implementation steps: Given a training data set Ω = {(*x*_*i*_, *t*_*i*_)|*x*_*i*_ ∈ *R*^*n*^, *t*_*i*_ ∈ *R*^*m*^, *i* = 1, 2, ⋯, *N*}, the hidden layer and output functions *G*(*α*_*i*_, *b*_*i*_, *x*), the number of hidden nodes is *L*.

Step 1: Initialize phase: Select a partial dataset $\Omega_{0}={\left\{\left(x_{i},t_{i}\right)\right\}}_{i=1}^{N_{0}}$ from $\Omega_{0}={\left\{\left(x_{i},t_{i}\right)\right\}}_{i=1}^{N_{0}},N_{0}\geq L.$

1. Randomly select the input weights *α*_*i*_ and the bias *b*_*i*_, *i* = 1, ⋯, *L*.
2. Calculate the hidden layer output matrix *H*_0_, there
  $$H_{0}={\left(\begin{array}{ccc} G(\alpha_{1},b_{1},x_{1}) & \cdots & G(\alpha_{L},b_{L},x_{1})\\ G(\alpha_{1},b_{1},x_{2}) & \cdots & G(\alpha_{L},b_{L},x_{2})\\ \begin{array}{c} \vdots \\ G(\alpha_{1},b_{1},x_{N_{0}}) \end{array} & \begin{array}{c} \;\\ \cdots \end{array} & \begin{array}{c} \vdots \\ G(\alpha_{L},b_{L},x_{N_{0}}) \end{array} \end{array}\right)}_{N_{0}\times L}$$ (7)
3. Calculate the initial output weights $\beta^{0}=P_{0}H_{0}^{T}T_{0}$, there $P_{0}={\left(H_{0}^{T}H_{0}\right)}^{-1},\;T_{0}={\left(t_{1},t_{2},\cdots ,t_{N_{0}}\right)}^{T}$.
4. set *k* = 0.

Step 2: Sequence learning stage: Suppose that the data block added is $\Omega_{k+1}={\left\{\left(x_{i},t_{i}\right)\right\}}_{i=(\sum_{j=0}^{k}N_{j})+1}^{\sum_{j=0}^{k+1}N_{j}}$ in *k* + 1 step, there *N*_*k*+1_ indicates the number of data added in step *k* + 1.

1. Compute the hidden layer output matrix *H*_*k*+1_ for newly added data, there
  $$H_{k+1}=\left(\begin{array}{ccc} G\left(\alpha_{1},b_{1},x_{\left(\sum_{j=0}^{k}N_{j}\right)+1}\right) & \cdots & G\left(\alpha_{L},b_{L},x_{\left(\sum_{j=0}^{k}N_{j}\right)+1}\right)\\ G\left(\alpha_{1},b_{1},x_{\left(\sum_{j=0}^{k}N_{j}\right)+2}\right) & \cdots & G\left(\alpha_{L},b_{L},x_{\left(\sum_{j=0}^{k}N_{j}\right)+2}\right)\\ \vdots & & \vdots \\ G\left(\alpha_{1},b_{1},x_{\sum_{j=0}^{k}N_{j}}\right) & \cdots & G\left(\alpha_{L},b_{L},x_{\sum_{j=0}^{k}N_{j}}\right) \end{array}\right)$$ (8)
2. set $T_{k+1}={\left(t_{(\sum_{j=0}^{k}N_{j})+1},t_{(\sum_{j=0}^{k}N_{j})+1},\cdots ,t_{(\sum_{j=0}^{k}N_{j})+1}\right)}^{T}$.
3. Calculate the output weights *β*^k+1^, there $\beta^{k+1}=\beta^{k}+P_{k}H_{k+1}^{T}(T_{k+1}-H_{k+1}\beta^{k})$
  $$P_{k+1}=P_{k}-P_{k}H_{k+1}^{T}{\left(I+H_{k+1}P_{k}H_{k+1}^{T}\right)}^{-1}H_{k+1}P_{k}$$ (9)
4. set *k* = *k* + 1, return to step 2.

When *N*_0_ = *N*, OS-ELM algorithm is equivalent to the original ELM algorithm, OS-ELM algorithm can not only learn data one by one but also to learn data block by block and abandon the data which have been studied immediately after the end of learning.

### Self-fitting OS-ELM

In sequential learning, some partial training data arrives in time sequential fashion: {(*x*_(0)_, *t*_(0)_), (*x*_(1)_, *t*_(1)_), ⋯, (*x*_(*k*)_, *t*_(*k*)_)}, Learning is the process of constructing function $\hat{\beta }$ to map between observation and its nature called (class). When the number of training data *N* → *∞*, we need to address the expected value of $\beta_{\infty }=\hat{\beta }$.

Learning from the data *Ω*_*n*_ is the process to select a function *β*_*n*_ from a class of B by minimizing the empirical squared error $e_{n}(\beta )=(1/n)\sum_{i=1}^{n}{\left(H_{i}\beta -T_{i}\right)}^{2}$ with the error probability $L(\beta_{n})=P\{I_{\left\{H\beta_{n}\right\}}\neq T|\Omega_{n}\}$ of the resulting classifier. The empirical squared error minimization is consistent under general conditions.

Based on Law of Large Numbers (LLN) theorem, we can make sure that the consistency of expected value of learning model is Eq (6) $\hat{\beta }=H^{+}T$, in sequential learning with the number of training data $\hat{\beta }=H^{+}T$.

### Intrusion detection model based on OS-ELM

#### Data preprocessing phase

In this model, the original data is firstly preprocessed and partial data of the original data set are randomly selected as the sample data set due to take into account a large amount of original data, the character data in the data set is transformed into Digital data and then normalize the data. After the processing of the original data is completed, considering the time complexity and computational efficiency, we will use the Gain Ratio Evaluation method to reduce the dimension of the experimental data. Gain ratio evaluation is a filtering feature selection method based on information metrics and information gain is the most important and useful feature of data selection. The information gain calculates the importance of information on the amount of information brought about by each feature before and after information is added to the data set and assesses the significance of the feature to the entire data.

Let *X*, *Y* be random variables, the information entropy of *X* and the entropy of *Y* with regard to *X* are defined as:

$$H(X)=-\sum_{i}P(x_{i}){\text{log}}_{2}\;\left(P(x_{i})\right)$$ (10)

$$H(X|Y)=-\sum_{j}P(y_{i})\sum_{i}P(x_{i}|y_{i}){\text{log}}_{2}\;\left(P(x_{i},y_{i})\right)$$ (11)

Information gain is expressed as the difference of information entropy, defined as:

InformationGain(X,Y)=H(X)-H(X|Y) (12)

Eq 12 can also be written as:

$$InformationGain\left(Ex,a\right)=H\left(Ex\right)-\sum_{v\in values\left(a\right)}\left(\frac{\left|x\in Ex|value\left(x,a\right)=v\right|}{\left|Ex\right|}\cdot H(\{x\in Ex|value\left(x,a\right)=v\})\right)$$ (13)

Information gain calculates the category appears in the category of information gain for a feature, that is feature before and after the appearance of information entropy difference in the feature selection. The greater the information gain of a feature, the more important its contribution to the taxonomy. But the information gain method is biased from a large number of attribute values to select the appropriate property and lead to over-fitting easily. Therefore, we use the gain ratio and intrinsic information extraction method. Eq 14 reflects the entropy of all sample probability distributions, and Eq 15 reflects the relationship between the gain ratio and the information gain.

$$IntrinsicValue(Ex,a)=-\sum_{v\in values(a)}\left(\frac{\left|x\in Ex|value(x,a)=v\right|}{\left|Ex\right|}∙{\text{log}}_{2}\;\left(\frac{\left|x\in Ex|value(x,a)=v\right|}{\left|Ex\right|}\right)\right)$$ (14)

$$GainRatio(S,A)=\frac{InformationGain(Ex,a)}{IntrinsicValue(Ex,a)}$$ (15)

#### Initialization phase

Firstly, a part of data set is selected as training data set to initialize the network, and the number of hidden nodes is set to L, then the input weight and bias value of hidden layer nodes are selected randomly, calculate the hidden layer output matrix and initial output weight Value to complete the initialization phase.

#### Online sequence learning phase

**Fig 2:**
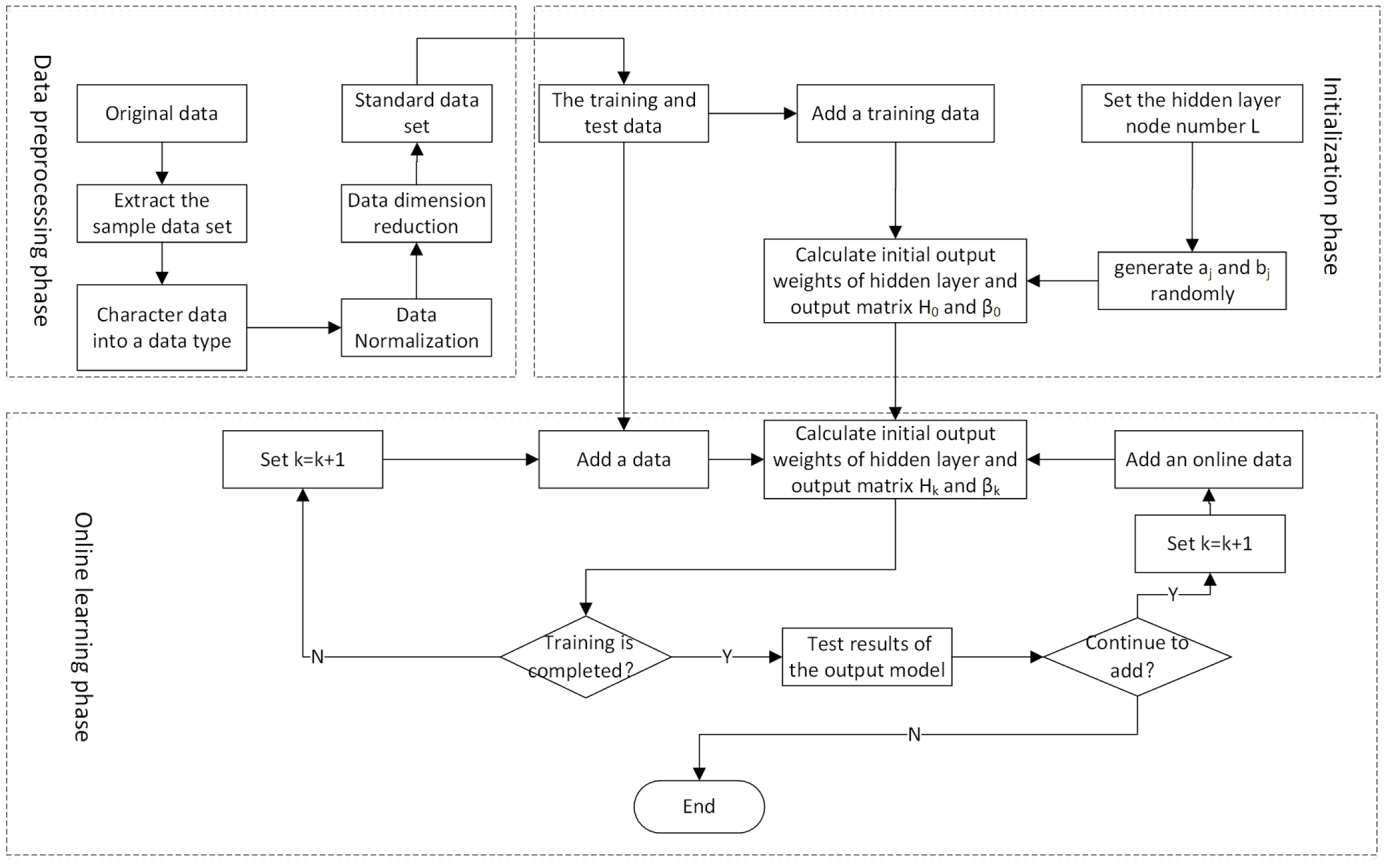
Flow chart of intrusion detection model based on OS-ELM.

Given the kth data segment, update the hidden-layer output matrix and the initial output weight, set k = k + 1, then return to the previous iterative update process. Test on the training data if the current training data training is completed and output the accuracy, the output of the initial value of the hidden layer, If the data is added during the training process, the update process will be repeated until all the training data have been trained, thus completing the online learning phase. The specific process is shown in Fig 2:

## Experimental results and analysis

### Analysis of experimental data sets

The evaluation utilizes a data-set from Advanced Metering Infrastructure (AMI) contain up to 33 million lines of data. This data-set corresponds to 1-day worth of data on August 1, 2012 with up to 2373 smart meters [31], We can get the data set from http://www.ucd.ie/issda/data/commissionforenergyregulationcer/.

In general, smart meter data are in the form of time series and are arranged in such a way that they appear as tuples. The smart meters that record samples every 15 minutes and every 1 minute have been identified, the data file format is as shown in Table 1:

**Table 1:** Format for the smart meter data.

Attribute	Definition
Meter Number	Unique smart meter identification number
Date	Date with the following format ‘YYYYMMDD’
Data	A matrix of NoOfPointsPerDay-by-NoOfChannels
	The NoOfPointsPerDay will be 96 for a resolution of 15 minutes, or 1440 for a resolution of 1 minute. The NoOfChannels will be 2 or 20

The model was trained ten times and each time used random sampling and the training data is composed of 120000 samples and the testing data compose of 20000 samples of records which is randomly selected from the complete data set.

### Evaluation indicators

Intrusion detection evaluation from the performance point of view to verify the effectiveness of the intrusion detection method and feasibility of this article using the accuracy rate, false positive rate and false negative rate, training time and test time five indicators.

1. TN (True Negative) Indicates the number of which normal data is correctly recognized as normal data.
2. FN (False Negative) Indicates that number of which normal data is recognized as an alarm.
3. TP (True Positive) Indicates the number of which alarm is correctly recognized as an alarm.
4. FP (False Positive) Indicates the number of which alarm is recognized as normal data

Therefore, three definitions of indicators can be described as follows:

1. Accuracy Rate: $AccuracyRate(AR)=\frac{TN+TP}{TN+FN+TP+FP}$, the higher the accuracy rate, the better the algorithm.
2. False Positive Rate: $FalsePositiveRate(FPR)=\frac{FP}{TN+FP}$, the lower the false positive rate, the better the algorithm.
3. False Negative Rate: $FalseNegativeRate(FNR)=\frac{FN}{FN+TP}$, the lower the false negative rate, the better the algorithm.

### Feature selection

**Fig 3:**
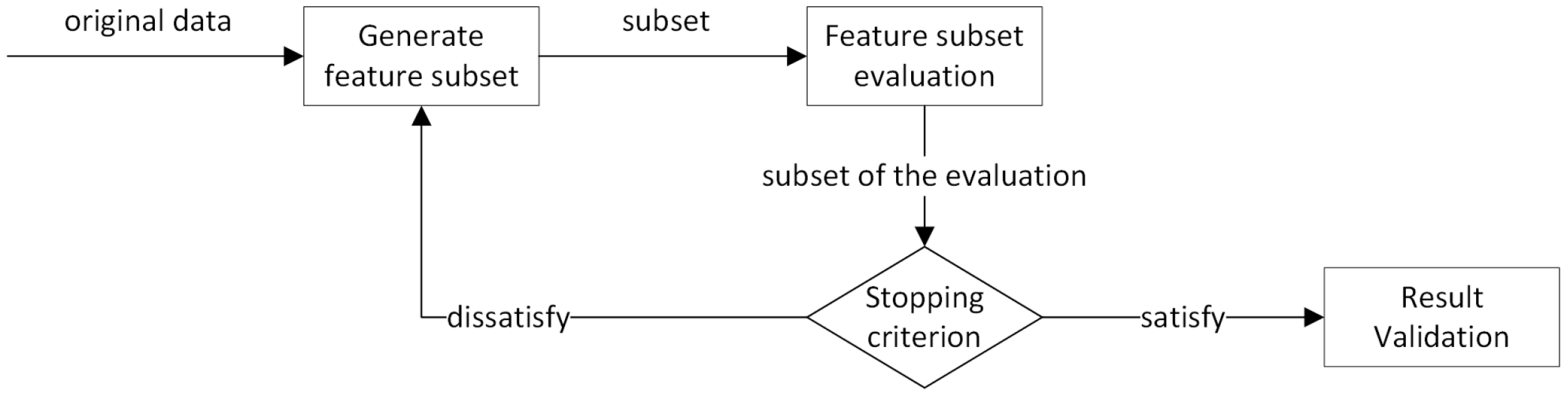
Flowchart of feature extraction.

In order to improve the efficiency of the detection system and reduce computational losses, we use the method of gain rate evaluation to characterize the data. A typical feature extraction process is shown in Fig 3:

According to the requirement of accuracy and computational complexity, an attribute value below the threshold value may be considered to have minimal influence and is removed in actual use. The use of dimensionality reduction data can effectively reduce the computational complexity and improve the performance of the algorithm. The gain ratio threshold is selected and the relevant experiments are performed in the subsequent experiments and the appropriate values are selected based on the experimental results.

### Experimental design and experimental results

#### Gain ratio threshold and feature selection

The main process of machine learning is to construct a classifier by using training data for a data sample and further classify the samples by classifier. However, it is not easy to deal with the data with high dimensionality, and the time complexity of the algorithm will increase with the increase of the dimension. So we need to use the method of reducing the feature dimension.

In order to validate the effectiveness and feasibility of the proposed method, we compare this method with Fisher, Relief, mRMR and InfoGain, which are the methods of feature selection in this paper.

**Fig 4:**
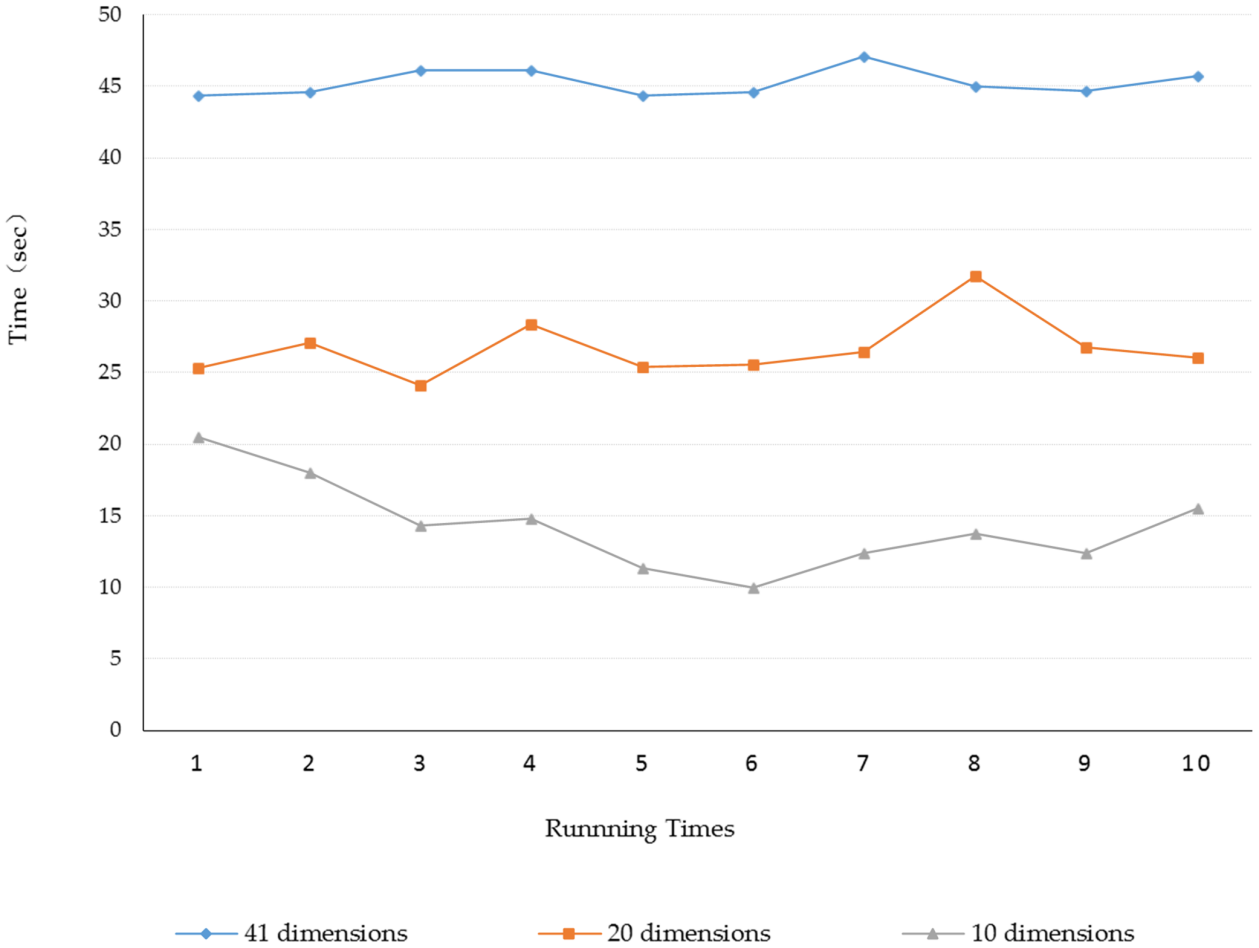
Execution time of the algorithm.

In the same system environment, the OS-ELM algorithm with the number of hidden nodes is 200, and the activation function selects sigmoid function and the initial block selection is 300, which use 2 fold cross-test method and repeat 10 experiments. It documents the accuracy of various classification algorithms and the running time of the algorithm under different dimensions. Experimental results are shown in Fig 4:

The abscissa represents the running times of the algorithm and the ordinate represents the execution time of the algorithm in Fig 4. From the training time curve of each dimension in the graph, we can see that the training time of the learning algorithm can be reduced effectively after the feather selection. The shorter the dimension, the shorter the training time. The following experiment will validate the accuracy of each algorithm in each dimension to help us select the appropriate dimension.

**Fig 5:**
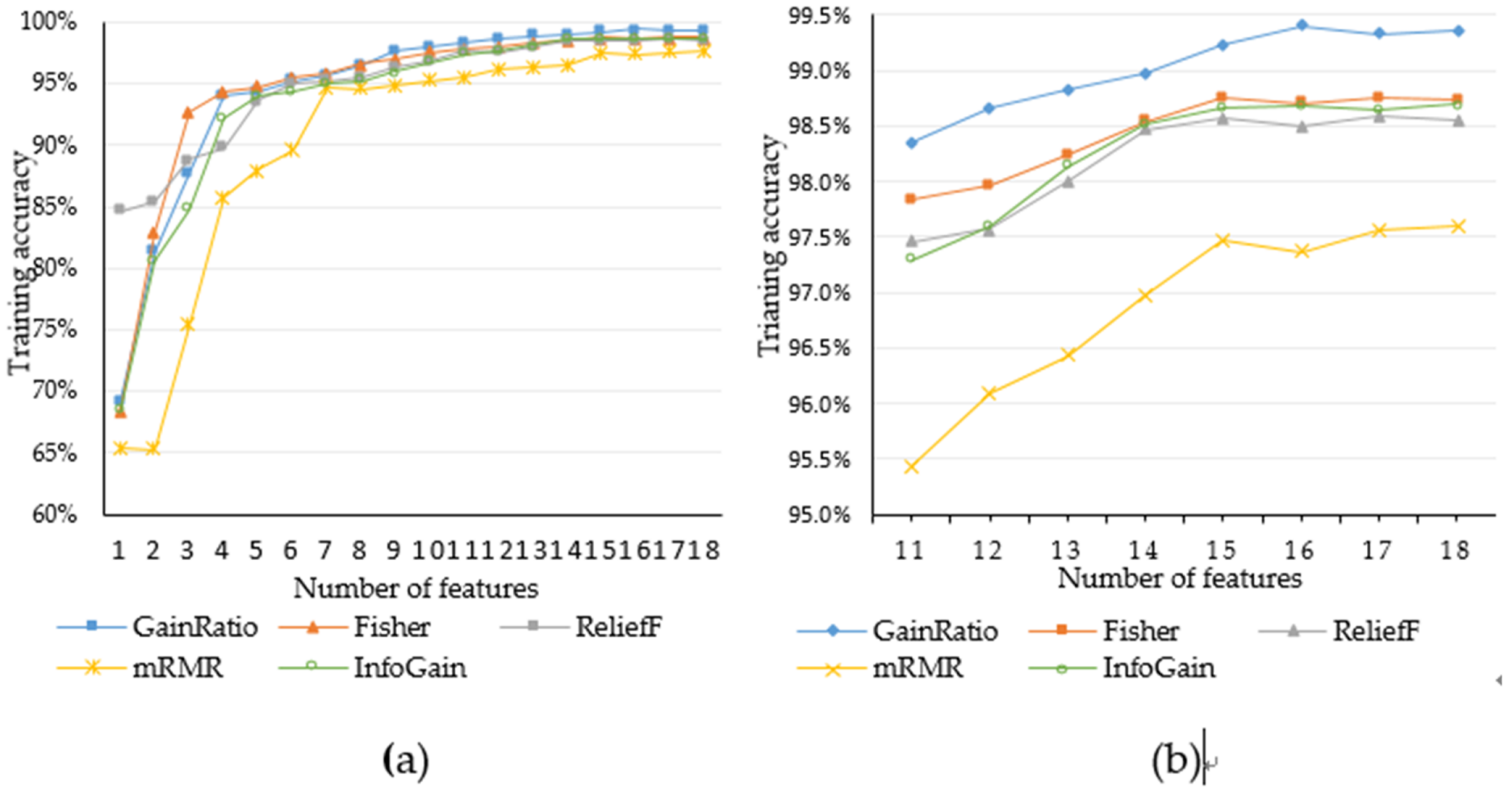
(a) Comparison of the accuracy rate of the classification algorithm and (b) The accuracy of 11 to 18 dimensions.

In the experiment, each algorithm was used to extract the experimental data, and then double cross-validation was used to measure the accuracy. Those are as shown in Fig 5:

**F**rom the above experiment we can see that the results of eighteen experiments that start from the first feature and the successive addition of a feature, until the sort of the first fifteen characteristics of the algorithm to join the results. It can be seen from Fig 5(a) that the accuracy of Gain Ratio, Fisher, ReliefF, and InfoGain begins to stabilize when the fifth feature is introduced. mRMR begins to stabilize after introducing the seventh feature. After the introduction of the tenth feature, the accuracy rate increases slowly with the increase of the feature number and the growth rate slows down. As can be seen from the Fig 5, when the number of features selected more than fifteen, the accuracy rate has stabilized, no significant growth. In order to facilitate the selection of the algorithm, we combine the ten features to the eighteen features in this interval as shown in Fig 5(b).

It can be seen that the accuracy of Gain Ratio is relatively high under the same feature number, which indicates that the Gain Ratio algorithm is more suitable for the OS-ELM algorithm. The accuracy can reach a satisfactory result when the subset of the feature is selected to fifteen features. Feature selection can effectively reduce the running time of the algorithm under the condition of guaranteeing the classification accuracy and it is very effective and feasible in intrusion detection. It can not only improve the efficiency of intrusion detection system but also the description of the attack is also significant. By defining key features, it can help to propose key indicators of specific attacks from different levels of attack description.

#### The parameters selection of OS-ELM

In the intrusion detection algorithm based on OS-ELM, the parameters are considered including the choice of excitation function, the number of hidden layer nodes, the number of initial nodes and the size of the block. The empirical parameter selection can only determine an approximate range, continue to compare experimentally and then adjust the final result to get an ideal parameter for this model when it is necessary. The following experiments are carried out based on the above.

**Fig 6:**
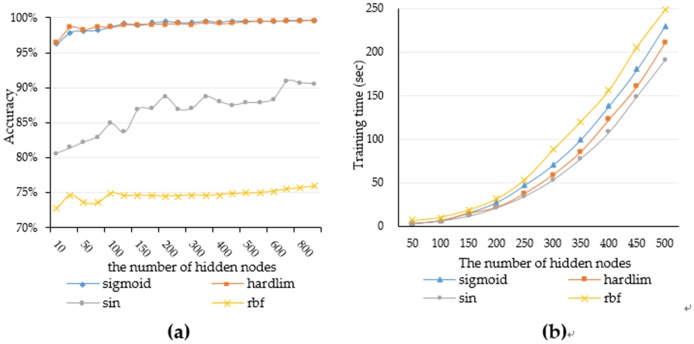
(a)The accuracy of different hidden node numbers and (b) the training time of different hidden node numbers.

**A. The choice of activation function**: Four different activation functions including the ‘sin’, ‘RBF’, ‘sigmoid’ and "hardlim" functions are used in this experiment. Fig 6(a) shows how the accuracy varies with the number of hidden nodes in the case of different excitation functions. We can see that the ‘RBF’ function has the worst performance under this experimental data set, the ‘sin’ function is not better than ‘hardlim’ and ‘sigmoid’ function. The accuracy of "hardlim" and "sigmoid" functions is similar to high precision, but the "hardlim" function is more stable than the "sigmoid" function. The training time of the ‘RBF’ function is the longest one compared with other functions and the training time of ‘sin’ function is least, but the accuracy rate is not the same as other functions. Fig 6(b) shows the change between training time and a different number of hidden nodes. The training time of the ‘hardlim’ function is shorter than the ‘sigmoid’ function and the curve of the ‘hardlim’ function training time grows smoother and more stable than the ‘sigmoid’, which is with the increase of the number of hidden nodes. So in the follow-up experiments, we will choose ‘hardlim’ function as the activation function.

**B. Selection of the number of blocks**: The original ELM algorithm uses a batch mode, that is, all the data are transmitted to the system in each training process. Adding one or a batch of data to the system in the OS-ELM method obviously will be more suitable for real-time intrusion detection. So this experiment will verify the number of blocks selected.

As it can be seen from Table 2, the sequential operation of 1–1 takes the highest time while batch the shortest time and any block mode (10–10,20–20, (10,30)) falls in between. If the block size is large, it approaches the time taken for batch mode operation. OS-ELM method is equivalent to ELM method when the size of the selected block is as the same as the original training sample size, the accuracy and applicability of ELM, which is lower than OS-ELM.

**Table 2:** The influence of block numbers.

Type of blocks	Accuracy	Testing time(s)	Training time(s)
batch	98.75%	0.3914	6.697
1–1	99.19%	0.3833	604.567
10–10	99.31%	0.3691	61.789
20–20	99.22%	0.3901	35.312
(10, 30)	99.25%	0.3875	31.243

This is because the OE-ELM algorithm introduces the idea of sequence learning, and the data can be added to the network piece by piece, and the original data will be discarded and not used after the completion of the study. The ELM algorithm will put the new data and put the data together to retrain the network, when new data is added to the network. If OS-ELM does not use the sequence of ideas to add a piece of data, then it is equivalent to the ELM algorithm. We choose OS-ELM because all the data is not a one-time added to the network in practice, and ELM will re-train the network in this environment, consume a lot of computing resources. As the amount of data increases and runtime increases, computing resource consumption and training time will increase. So the OS-ELM in the application is more in line with the actual needs of the situation. However, the block size and the overall training time are inversely proportional to the trend, that is because the more block size, the larger training data set and needs more RAM space. When this space exceeds a limit, the procedure slows down. So we consider the impact of the two on the experimental results and finally we choose the size of each block is 20 data.

**C. The choice of the number of initial values**: The first step is to determine the number of initial values in the above-mentioned initialization phase. The number of initial values are due to different processing problem and the initial values were selected for proposed intrusion detection OS-ELM algorithm, we conduct experiment in different initial value numbers, the experimental results (L = 100, L = 200, L = 300) are shown in the Tables 3, 4 and 5.

**Table 3:** The influence of the initial value numbers (L = 100).

Numbers of hidden nodes	Numbers of initial values	Training time(s)	Accuracy	Proportion of implied points
100	100	6.568	97.97%	100%
100	150	6.674	98.16%	67%
100	200	6.586	98.19%	50%
100	300	6.427	98.51%	33%
100	400	6.415	98.53%	25%
100	500	6.378	98.66%	20%
100	700	6.357	98.75%	14%
100	900	6.345	98.74%	11%
100	1000	6.389	98.87%	10%

**Table 4:** The influence of the initial value numbers (L = 200).

Numbers of hidden nodes	Numbers of initial values	Training time(s)	Accuracy	Proportion of implied points
200	200	24.524	98.66%	100%
200	250	23.739	98.82%	80%
200	300	23.756	99.01%	67%
200	400	23.057	99.09%	50%
200	500	22.797	99.17%	40%
200	700	22.554	99.28%	29%
200	900	21.767	99.41%	22%
200	1200	21.482	99.45%	17%
200	1500	21.353	99.47%	13.33%
200	2000	21.044	99.63%	10.00%

**Table 5:** The influence of the initial value numbers (L = 300).

Numbers of hidden nodes	Numbers of initial values	Training time(s)	Accuracy	Proportion of implied points
300	300	62.456	99.21%	100.00%
300	400	62.826	99.23%	75.00%
300	500	63.044	99.46%	60.00%
300	600	63.546	99.51%	50.00%
300	750	63.796	99.56%	40.00%
300	1000	62.158	99.58%	30.00%
300	1200	62.386	99.67%	25.00%
300	1500	61.164	99.74%	20.00%
300	2000	60.964	99.74%	15.00%
300	3000	60.235	99.76%	10.00%

It can be seen from the above three experiments that in terms of accuracy, the degree of influence that the absolute difference of the number of implicit nodes and initial values is higher than the degree of influence that the number of implicit nodes and initial values in the accuracy aspect. When the initial value is greater than the number of hidden nodes, the performance of the OS-ELM intrusion detection system proposed is close to optimal.

#### Performance comparison of OS-ELM algorithm with other algorithms

In order to verify the effectiveness of the proposed detection system, this section will compare the experimental performance of various algorithms including BP neural network, radial basis function (RBF) network, extreme learning machine (ELM) and online sequence extreme learning machine (OS-ELM). Experimental parameters for training time, accuracy, false positive rate, false negative rate and experimental parameters select the default parameters, the use of 10 times the cross-validation method. The experimental results are shown in the Table 6.

**Table 6:** The results of different learning algorithms.

	Training time(s)	Accuracy	FPR	FNR
OS-ELM	59.474	97.239%	5.897%	3.614%
ELM	7.641	95.369%	6.067%	4.397%
BPNN	343.439	95.647%	4.374%	4.594%
RBF	99.793	96.034%	8.144%	2.831%

**Fig 7:**
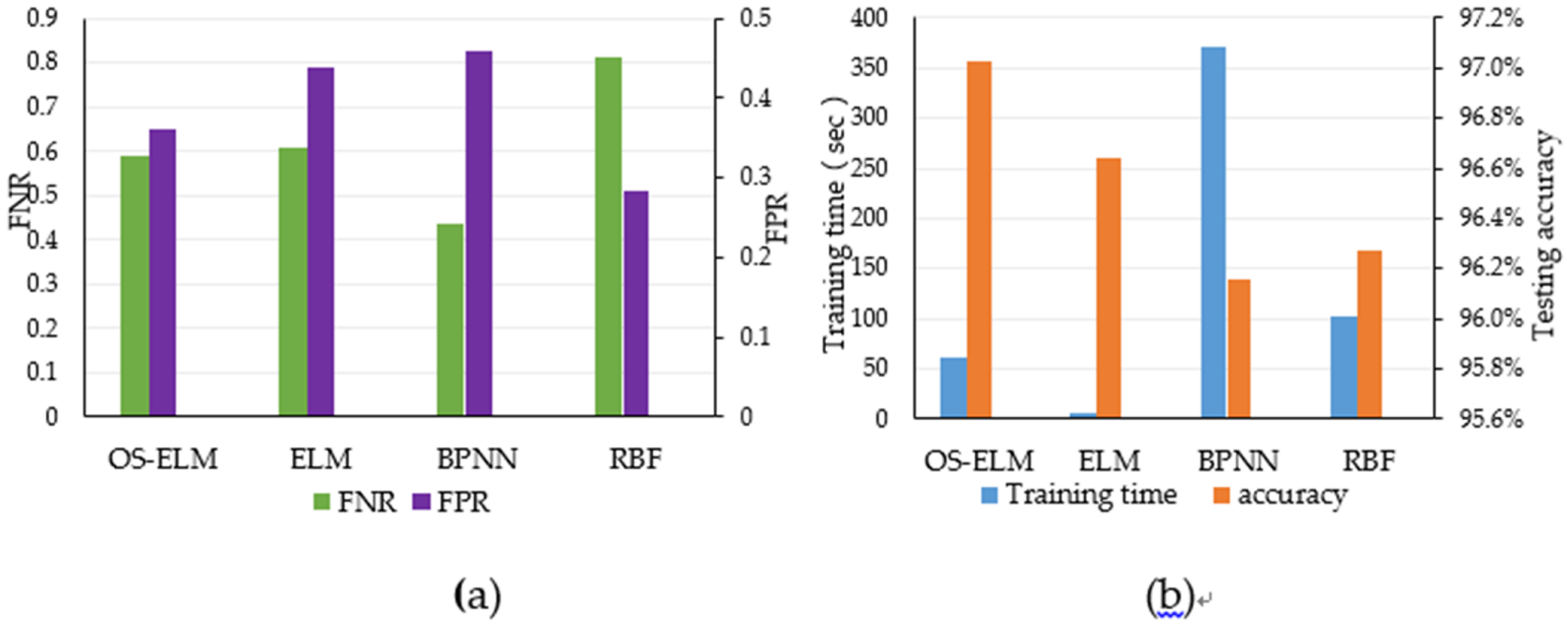
The results of for the four compared algorithms. (a) The FNR and FPR Comparison of algorithms. And (b) the Performance Comparison of Algorithms.

It can be seen from Fig 7(a) and 7(b) that the OS-ELM is faster and more accurate than the BP neural network and RBF radial basis network in training time. Compared with the traditional extreme learning machine, the data input of the intrusion detection system improves the accuracy, false positive rate, and false negative rate are improved and OS-ELM is more effective compared with the batch mode of other algorithms batch mode in the data input in intrusion detection systems.

## Conclusion

In this paper, we propose an intrusion detection system model based on the online sequential extreme learning machine for advanced measurement infrastructure. In the experiment, we use the gain ratio evaluation method to reduce the dimension of the sample dataset. The OS-ELM algorithm is used to classify and train datasets. Then a large number of experiments are conducted to select the optimal algorithm parameters for the proposed system. Finally, the proposed OS-ELM-based intrusion detection system is compared with other similar algorithms and the experimental results verify the effectiveness and feasibility of the proposed method.
